# RCCC_Pred: A Novel Method for Sequence-Based Identification of Renal Clear Cell Carcinoma Genes through DNA Mutations and a Blend of Features

**DOI:** 10.3390/diagnostics12123036

**Published:** 2022-12-03

**Authors:** Arfa Hassan, Tamim Alkhalifah, Fahad Alturise, Yaser Daanial Khan

**Affiliations:** 1 Department of Computer Science, School of Systems and Technology, University of Management and Technology, Lahore 54770, Pakistan; 2 Department of Computer, College of Science and Arts in Ar Rass, Qassim University, Ar Rass 58892, Qassim, Saudi Arabia

**Keywords:** clear cell renal carcinoma, cancer driver mutations, machine learning, prediction, statistical moments

## Abstract

To save lives from cancer, it is very crucial to diagnose it at its early stages. One solution to early diagnosis lies in the identification of the cancer driver genes and their mutations. Such diagnostics can substantially minimize the mortality rate of this deadly disease. However, concurrently, the identification of cancer driver gene mutation through experimental mechanisms could be an expensive, slow, and laborious job. The advancement of computational strategies that could help in the early prediction of cancer growth effectively and accurately is thus highly needed towards early diagnoses and a decrease in the mortality rates due to this disease. Herein, we aim to predict clear cell renal carcinoma (RCCC) at the level of the genes, using the genomic sequences. The dataset was taken from IntOgen Cancer Mutations Browser and all genes’ standard DNA sequences were taken from the NCBI database. Using cancer-associated information of mutation from INTOGEN, the benchmark dataset was generated by creating the mutations in original sequences. After extensive feature extraction, the dataset was used to train ANN+ Hist Gradient boosting that could perform the classification of RCCC genes, other cancer-associated genes, and non-cancerous/unknown (non-tumor driver) genes. Through an independent dataset test, the accuracy observed was 83%, whereas the 10-fold cross-validation and Jackknife validation yielded 98% and 100% accurate results, respectively. The proposed predictor RCCC_Pred is able to identify RCCC genes with high accuracy and efficiency and can help scientists/researchers easily predict and diagnose cancer at its early stages.

## 1. Introduction

In human DNA sequences, change due to certain reasons is called a mutation [1,2]. Mutations can be good or bad and are used by scientists to study human health, body cell development, etc. Health scientists already have conducted a lot of work to identify mutations in human beings [3], because this identification could work as a foundation for personalized medicine [4]. Furthermore, genetic engineering can play an important role to prevent disease, as well as making an early diagnosis to control the death rate [5,6].

Gene mutations play a vital role in cancerous cell generation [7], while genetic engineering is capable to predict deadly diseases such as cancer even before the symptoms of the disease are produced in the human body [8,9]. For this purpose, many techniques are available, but the traditional method used is lab evaluation, which is time-consuming, as well as expensive [10,11]. Cancer is a disease that can affect all the different body systems and tissues in human beings. Malignancy of cancer has more than 100 types and, back in 2010 alone, cancer was responsible for 1 out of 8 deaths worldwide [12]. Kidney malignancy is one of the different types of malignant cancers, but it is not a single disease, actually, it is a combination of many types, for example, clear cell, type 1 papillary, type 2 papillary, chromophobe, TFE3, TFEB, and oncocytoma [13].

Among all these types, the prevalence of renal cell carcinoma (RCC) or kidney cancer is all over the world, but most of the cases are reported in North America. Gender and age play an important role in the occurrence of the disease, as males above 65 years old are considered to be more prone to this disease, and obesity is also one of the main causes of the disease [14,15]. It initially starts from the outer walls of the kidney [16]. Based on some specific characteristics, RCC is divided into three main types of RCC. Clear cell RCCs—these are the most usual type of renal cell carcinoma. Researcher call them clear cells because most of the time cells within the tumor are clear. Papillary cell RCCs—these are the second most common type of kidney cancer. Papillary cells are distinguished by small, rounded protuberances on their surface. These tumors present as either Type 1 or the more aggressive Type 2 forms. Chromophobe cell RCCs—these are the third most common form of kidney malignancy. Scientists call them chromophobe because these cells do not acquire colored stains easily [17].

Various studies have been reported previously which targeted the prediction of cancer-driver gene mutations. Luo, in 2019, proposed deepdriver, which used a deep convolution neural network approach for cancer driver mutation prediction. The proposed method can predict breast and colorectal cancer. The AUC scores of deepdriver on cancer and colorectal cancer are 0.98 and 0.97, respectively [18]. In 2018, Wand et al. proposed a Bayesian hierarchical modeling algorithm for cancer driver mutation prediction named rDriver. They examined 3080 samples of 8 different kinds of cancer, in which the rDriver predicted 1389 affected samples. The evaluation process of the rDriver predictions method is conducted by using engineered cell line models and gives good results. The value results are a positive predictive value of 0.94 in PIK3CA genes [19].

In 2017, Pi-Jung et al. proposed a CNV method for cancer driver mutation prediction. For simulation and results, they used four TCGA datasets (BRCA, HNSC, KIRC, and THCA). They covered breast, head, neck, thyroid, and kidney cancer genes. They also discovered rare driver genes in their work. They did all the work with the help of gene sequence length [20]. A driver mutation provided growth advantages to the affected tumor cell. In 2013, Mao et. Al. introduced the candrA tool for the prediction of cancer driver mutations. The proposed method was based on a set of 95 structural and evolutionary features by using 10 functional prediction algorithms such as CHASM, SIFT, and Mutation Assessor. They used two mutation datasets, GBM and OVC [21].

The majority of the work carried out for the identification of RCCC driver genes uses experimental lab procedures, and those who used computational approaches lacked in performance due to fewer data, such as Kocak et al. [22]. The focus of their study was only on the *PBRM1* gene, and the sample size for training was also not good. Thus, to address this problem, the present study aims to propose a prediction model for renal clear cell carcinoma mutations in gene sequences using machine learning algorithms. We curated a comparatively large dataset from the IntOgen and NCBI databases, and after meticulous and thorough feature extraction, we trained different machine learning classifiers. After an exhaustive evaluation of machine learning classifiers, the best-performing classifier was selected as the final model and was compared with existing methods. The proposed method is easy to use, efficient and accurate for obtaining results, scientists and researchers only need sequence information and can avoid hectic experimentations. To do so, the intended clinical use is that scientists, researchers, and others in the clinical community can opt for a system developed based on the proposed method and obtain results by inputting the gene sequence. The sequence could be from human biological samples. The system will help them classify it as a potential RCCC gene or not.

## 2. Materials and Methods

**Figure 1:**
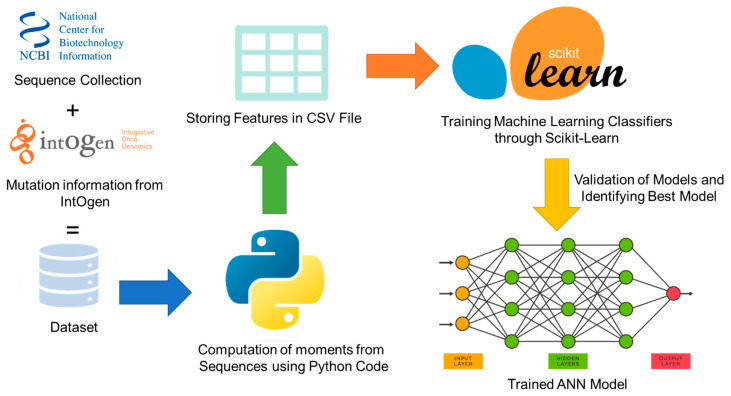
Architecture diagram of the proposed model.

A method named RCCC_Pred is proposed in the present study for the identification of renal clear cell carcinoma driver genes and associated mutations. The detailed graphical representation of the proposed method is shown in Figure 1. Creating a benchmark dataset from raw data is a great challenge for this work, considering the importance of human life and health.

### 2.1. Dataset Collection and Pre-Processing

As human health is a sensitive issue, datasets for human-health-related application needs perfection and accuracy. For this research work, a raw dataset is collected from intogen_driver_mutations_catalog-2016.5 (https://www.intogen.org, Accessed on: 1 November 2021). This raw dataset contains basic information such as genes name, ID, position (of the gene on which mutation occurs), which nucleotide is mutated, and which new nucleotide is replaced. The standard sequence of the nucleotide sequence is taken from NCBI (https://www.ncbi.nlm.nih.gov/, Accessed on: 10 November 2021). To construct the benchmark dataset, the system takes an input of mutation from a CSV file and then updates the sequence file and saves it in .txt format in a specific folder. At the end of the process, the folder had approximately 11,000 samples, and every sample showed a unique entry. This raw dataset contained 26,000 unique entries of 11,000 different kinds of genes, out of which 4529 samples were selected for feature extraction after redundancy removal through CD-HIT [23], using the threshold of 0.7. The independent test dataset in the present study was created by using the train–test split. Here, we split the original data into a 70:30 ratio, i.e., 70% of the original data were used for training the model, while for testing, the remaining 30% was used.

### 2.2. Feature Extraction

After obtaining the raw data, thorough and meticulous feature extraction was performed for gene sequences to obtain the quantitative description of the data [24]. The system performs two approaches for feature extraction by using statistical moments [25]. Gene sequence data are position-sensitive data, so for the first layer of feature extraction the system uses position-sensitive raw, Hahn, and central moments [26]. Additionally, the results are saved into separate files. In the second layer of feature extraction, the first four statistical moments and maximum and minimum values are found and stored in a CSV file. The mathematical equation of the raw moment is described as [27]

(1) $$R_{ab}=\sum_{x=1}^{n}\sum_{y=1}^{n}x^{a}y^{b}\beta_{ab}$$

To calculate the raw moment, the sequence is formed into an X’ matrix of n*n dimension. The mathematical expression for the central moment is shown in Equation (2).

(2) $$\;C_{ab}=\sum_{x=1}^{n}\sum_{y=1}^{n}{\left(x-\bar{e}\right)}^{a}{\left(y-\bar{d}\right)}^{b}\beta_{xy}$$

The Hahn moment requires a square matrix for calculation as input, so the system uses the following equation to calculate the Hahn of *n* order polynomial [28,29,30].

(3) $$Ha_{n}^{x,y}\left(b,N\right)={\left(N+y-1\right)}_{n}{\left(N-1\right)}_{n}\sum_{Q=0}^{n}{\left(-1\right)}^{Q}\frac{{\left(-n\right)}_{Q}{\left(-b\right)}_{k}{\left(2N+y+x-n-1\right)}_{Q}1}{{\left(N+y-1\right)}_{Q}{\left(N-1\right)}_{Q}}Q!$$

The machine learning algorithm requires a low-dimensional dataset for classification. In this low-dimensional dataset, every row shows a unique entity. For this purpose, the proposed system covers all the unique files into a 1D vector by using statistical moments and storing them in CSV files. After all the calculations, the final dataset contains 6 features for each entity, which are provided in Supplementary Information S1. The statistical equation moment is [31]

(4) $$moment_{pq}=\frac{1}{n}\sum_{j-1}^{n}{\left(ha-\bar{ha}\right)}^{pq}$$

### 2.3. Classification

**Figure 2:**
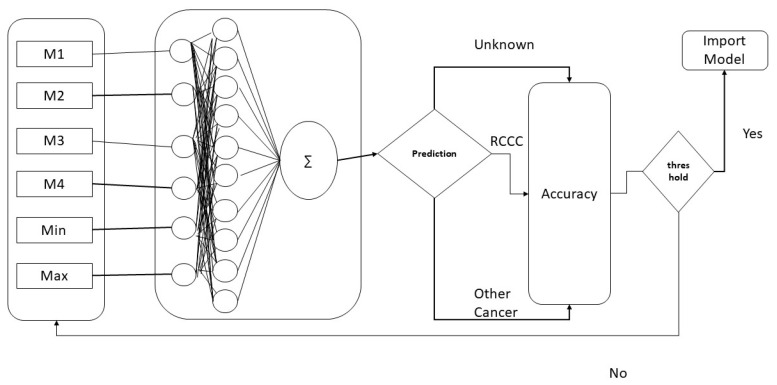
Architecture of the ANN model.

Using the featured vector extracted in the previous phase, a classifier is trained with the features from the dataset [32]. After feature extraction, 4529 unique entities are found, which are divided into three classes “kindey_tumor_driver”, “other_tumer_driver”, and “Unknown (non-tumour driver)”. A feed-forward artificial neural network (ANN) with backpropagation empowered by the hist gradient descent algorithm is used for prediction purposes [33]. ANN is an AI algorithm that is inspired by the human brain [34]. ANN takes inputs and combines them with the activation function and proceeds toward the output function. The representation of the ANN is explained in Figure 2.

Here, M1 to M4 represent the first 4 moments, while Min represents the minimum value of the computed moment and Max contains the maximum value of the computed moment for each sample. The mathematical equation of ANN is [35]

(5) $$ANN_{Q}=\sum_{j=1}^{n}W_{Qj}y_{j}$$

Gradient descent (GD) and adaptive learning methods are used for training the operational algorithm. GD is an optimization algorithm that is used to minimize the cost function and is very useful for MCC calculation. GD shows the best results in the analysis of data [24,36]. If the cost function of GD is [37]

(6) $$f\left(c,d\right)=\frac{1}{N}\sum_{i=1}^{n}{\left(y_{i}-\left(cz_{i}+d\right)\right)}^{2}$$

then the mathematical equation of GD is [38]

(7) $$f^{\prime }{}^{\left(c,d\right)}=\left[\begin{array}{c} \frac{df}{dm}\\ \frac{df}{dd} \end{array}\right]=\left[\begin{array}{c} \frac{1}{N}\sum^{\;}-2z_{i}\left(y_{i}-\left(mz_{i}+d\right)\right)\\ \frac{1}{N}\sum^{\;}-2\left(y_{i}-\left(mz_{i}+d\right)\right) \end{array}\right]$$

### 2.4. Model Evaluation

The most important part of any prediction model development is the accurate assessment of the model [39]. This accuracy evaluation is based on different factors. In this proposed work, various metrics were computed such as the specificity, sensitivity, and Matthew coefficient correlation (MCC) for the stability of the model and the accuracy of the model [33,40,41]. However, all these measures can be mathematically denoted as

(8) $$\mathrm{Specificity}=\frac{\mathrm{TN}}{\mathrm{TN}+\mathrm{FP}}$$

(9) $$\mathrm{Sensitivity}=\frac{\mathrm{TP}}{\mathrm{TP}+\mathrm{FN}}$$

(10) $$\mathrm{Accuracy}=\frac{\mathrm{TP}+\mathrm{TN}}{\mathrm{TP}+\mathrm{FN}+\mathrm{TN}+\mathrm{FP}}$$

(11) $$\mathrm{MCC}=\frac{\left(\mathrm{TP}*\mathrm{TN}\right)-\left(\mathrm{FP}*\mathrm{FN}\right)}{\sqrt{\left(\mathrm{TP}+\mathrm{FP}\right)\left(\mathrm{TP}+\mathrm{FN}\right)\left(\mathrm{TN}+\mathrm{FP}\right)\left(\mathrm{TN}+\mathrm{FN}\right)}}$$

In Equations (8)–(11), as described in [42], TP represents the true positive, i.e., the number of the data samples where the class label is positive, and the system predicts it is positive. TN represents the true negatives, which are the samples whose label was negative and the system also predicted as negative. FP is the false positive, representing negative samples, predicted incorrectly as positive by the predictor. Lastly, the FN are false negatives, representing positive samples predicted incorrectly as negative by the predictor [43]. As the description of these measures depicts, these are usually used for binary classification problems. However, in the present study, we had three different classes, i.e., RCCC, other tumors, and Unknown (non-tumor driver). Thus, to map our problem on these measures, we used the scheme shown in Table 1.

## 3. Results

In the proposed method, after data collection, the first step in the pre-processing layer was to perform the mutation and save the results. Later on, for all sequential data, features were computed and fed to machine learning classifiers for evaluation. For evaluating the training accuracy, self-consistency testing was performed in which the same training and testing data were used. To evaluate and validate the outcome of the prediction model, the proposed system was tested by using three different techniques, i.e., independent testing, K-cross-validation testing, and Jackknife testing.

### 3.1. Training Accuracy

The training accuracy was evaluated using self-consistency testing [44]. For this purpose, the same training and testing data were used. The evaluation scores are shown in Table 2.

Table 2 illustrate that the model was trained accurately, identifying all positive/negative samples correctly.

### 3.2. Validation of the Model through 10-Fold Cross-Validation and Jackknife Testing

The validation of results was performed using various evaluation metrics such as accuracy, specificity, sensitivity, and Matthews Correlation Coefficient. The test methods adapted were independent testing, K-cross-validation testing, and Jackknife testing [3].

**Figure 3:**
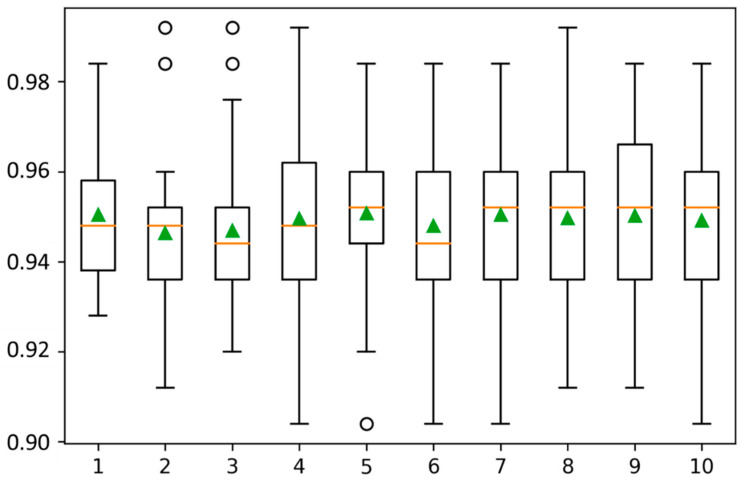
Accuracy scores of all 10 folds for 10-fold cross-validation. The circles represent outliers.

**Figure 4:**
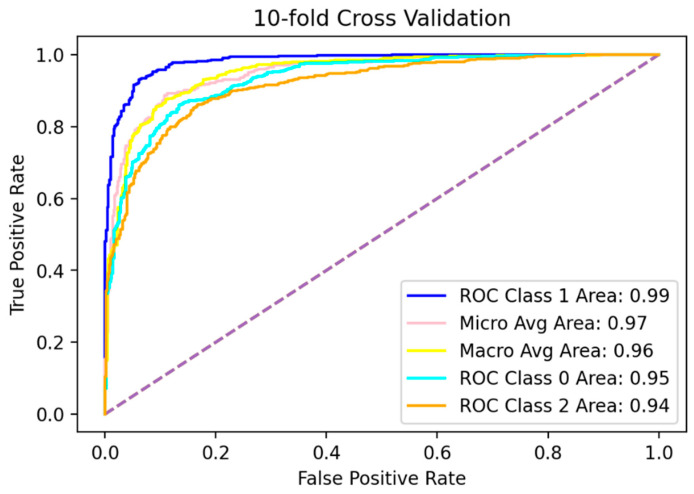
Mean ROC of 10-fold cross-validation.

In case of the unavailability of a separate test dataset, the best approach to test any predictive model is k-fold cross-validation [45]. Using k-fold cross-validation, the dataset is split into k-disjoint folds, and the model is validated k-times. In each iteration, k-1 folds are chosen for the training model, while the remaining 1-fold is used for testing. This testing fold is chosen separately in each iteration [46]. For the evaluation of the proposed model, the value of k was chosen as 10. The overall mean accuracy score is 0.98, and scores of the remaining measures, as well as for all folds, are shown in Table 3. The box plot graph is shown in Figure 3, while the ROC curves for all classes using 10-fold cross-validation are shown in Figure 4.

**Figure 5:**
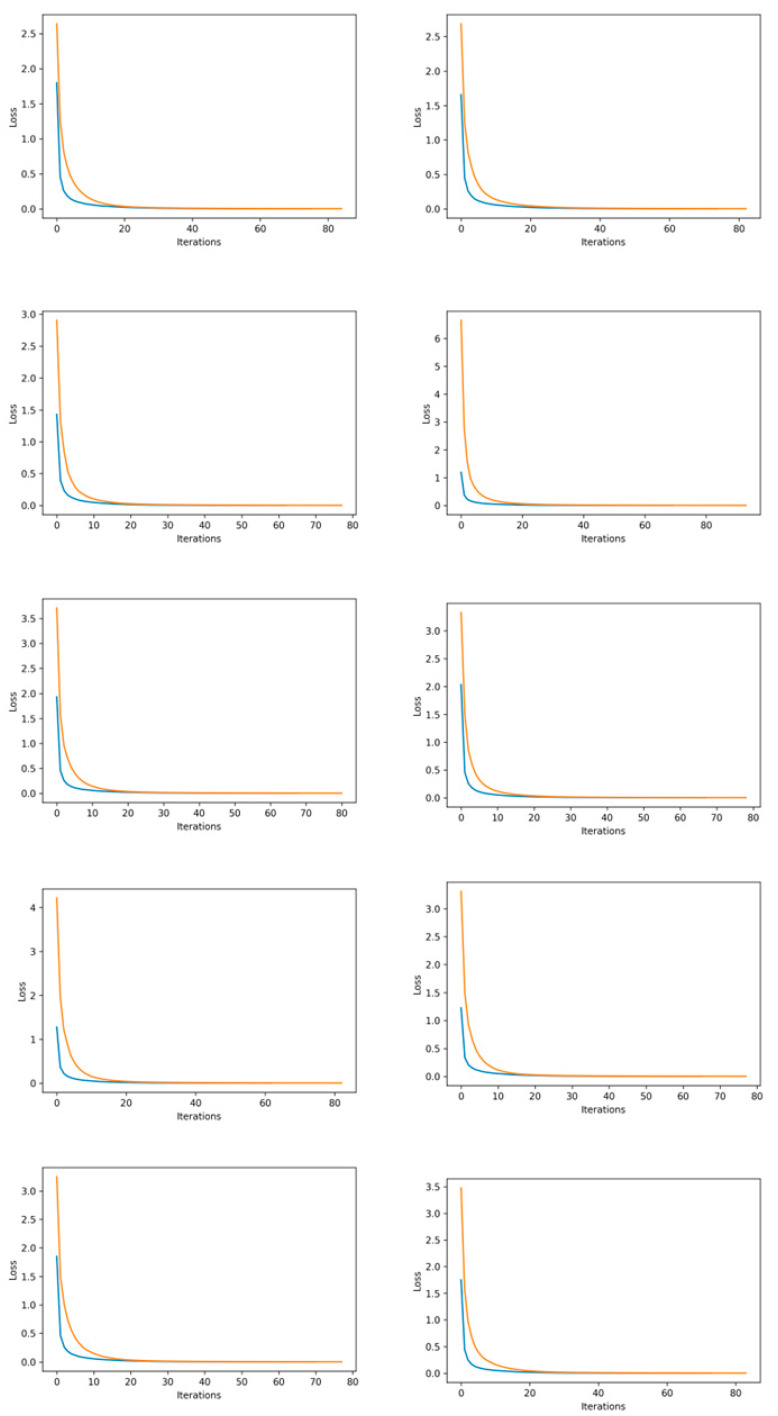
Training and validation curves for 10-fold cross-validation. Orange curves are for training, while blue curves are for validation.

To further elaborate on the 10-fold cross-validation, training and validation loss for each fold is shown in Figure 5.

In Figure 5, the curves are plotted between the number of iterations and losses. The neural network was trained till convergence by using an early stopping criterion, while max iterations were set as 3000. However, it was observed that for all 10 iterations of k-fold, the model converged on an average of 20–30 epochs. This is the reason that, after such epochs, we can observe that the loss curves converged at a point and turned into straight lines.

**Figure 6:**
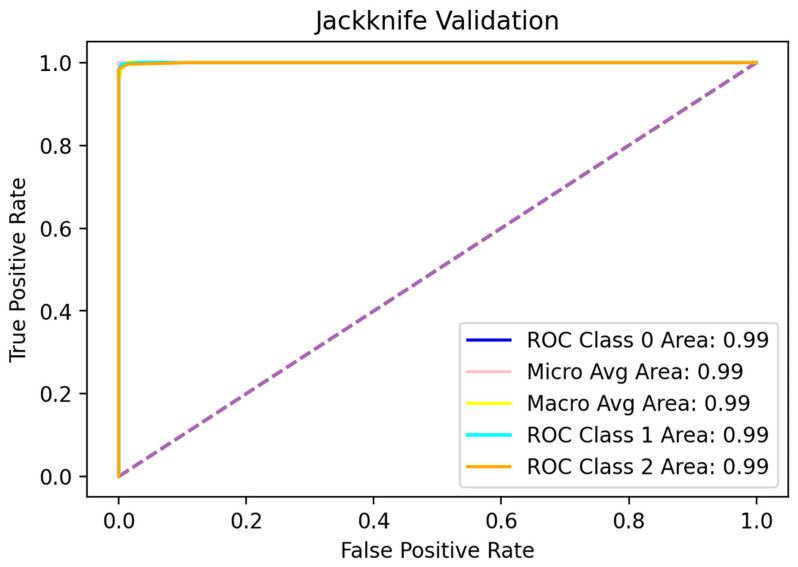
ROC curve for Jackknife validation.

For further exhaustive validation of the model, the Jackknife test was opted for. The Jackknife test is also referred to as Leave-One-Out cross-validation, and works on the same principle as k-fold cross-validation, with the value of k = the number of samples in the dataset [2]. It is known to be the least arbitrary method which can yield unique output for a given benchmark. After testing, the accuracy metrics were computed to evaluate the quality of the proposed algorithm. The results for Jackknife validation are shown in Table 4, while the ROC curve is shown in Figure 6.

Using the scores of Jackknife validation, RCCC_Pred was compared with a few existing methods. The results are shown in Table 5.

Herein, we compared the results of Jackknife testing of the present study with this method and observed a lack of performance. It could be observed that the proposed method outperformed the existing method by Kocak et al. [22] in terms of all accuracy measures.

### 3.3. Independent Dataset Validation

**Figure 7:**
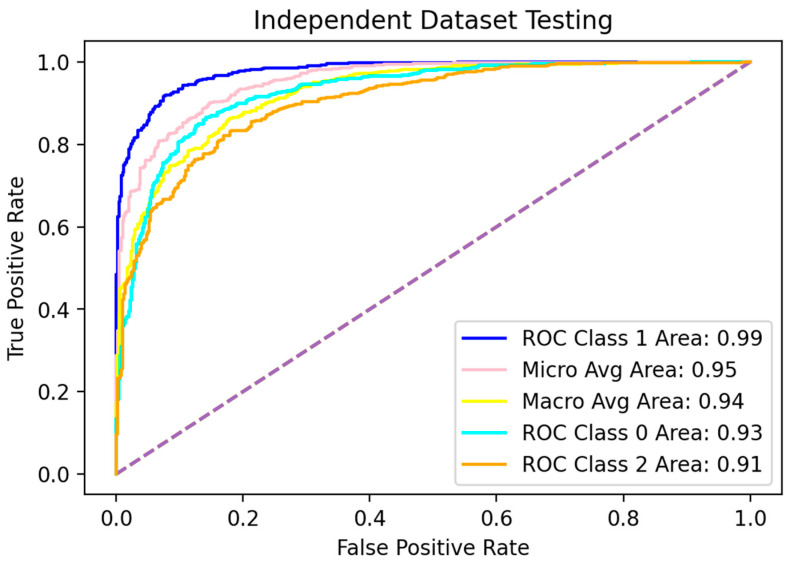
ROC curve of independent dataset test.

For any new predictor, it is of great importance to test its ability to predict against unknown data. Here, unknown data are referred to as the data which the model has not seen or observed during the training process [1,2,39]. Therefore, keeping in view the importance of this test, it was performed for the evaluation of the model proposed in the present study. As the whole dataset was created manually in this study, all possible data samples available at that time were already retrieved. Here, we trained the model from scratch using 70% of the data as described in the Methods while testing it for the remaining 30% of samples. The scores were computed along with the ROC curve and are represented in Table 6 and Figure 7, respectively.

### 3.4. Comparison with Other Classifiers

**Figure 8:**
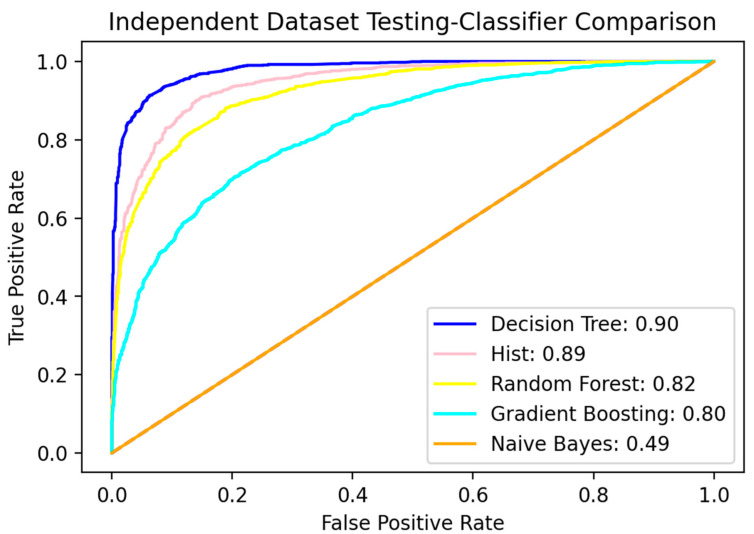
ROC curve of independent dataset test for all 5 classifiers.

Besides comparing the proposed method with previously existing methods, we have also run experiments to compare the performance of our method with different other classifiers. The benchmark dataset of the proposed method is also trained with other machine learning algorithms, but Hist Gradient boosting shows the best results. The details of other proposed models are shown in Table 7. Table 7 shows the accuracy scores for three different kinds of tests, which are Jackknife, independent, and cross-validation, while these tests are performed using five classifiers. To further elaborate on performance, the ROC curve for all five classifiers for the independent dataset testing is added in Figure 8.

The decision tree showed better performance in independent dataset testing, however, overall, the performance of Hist Gradient boosting was better as compared with the other classifiers. Based on these results, the Hist Gradient Boosting was considered as the final model for the proposed RCCC_Pred classifier.

## 4. Discussion

Herein, we proposed a prediction model named RCCC_Pred for renal clear cell carcinoma mutations in gene sequences using machine learning algorithms. We curated a dataset from IntOgen and NCBI databases and, after meticulous feature extraction, we trained different machine learning classifiers. After a thorough evaluation, the best-performing classifier was selected as the final model and was compared with existing methods. The decision tree showed better performance in independent dataset testing, however, overall, the performance of Hist Gradient boosting outperformed other classifiers. Based on these results, the Hist Gradient boosting was considered as the final model for the proposed RCCC_Pred classifier.

Previously, a few studies were proposed for renal clear cell carcinoma or other cancers [47]. A few machine-learning-based and experimental approaches have been proposed to study multicellular complexity and tissue specificity [48], as well as to study molecular interactions in cancer [49]. The majority of the work conducted for the identification of RCCC driver genes uses experimental lab procedures. A few research studies used AI-based approaches to predict cancer driver mutations, but their methods used a very limited amount of data. In a previous study reported by Kocak et al. [22], the researchers proposed a machine-learning-based algorithm for kidney cancer prediction at the level of the gene. The sample set for the machine learning consists of 161 label examples of augmented data, from which 74 mutations are recorded in the PBRM1 gene, and the other 87 occurred outside the PBRM1 gene. The focus of the study is only on one gene code, which is *PBRM1*, and the sample size for training is also not good. Due to the tiny dataset, the possibility of overfitting occurrence is very high, which affects the accuracy of the system.

In 2020, Kocak et al. [50] further extended their work by considering BAP1 mutation in clear cell renal cell carcinoma. However, again, the dataset was limited, comprising only 65 samples. Here, authors used a Random Forest classifier and correctly classified samples with 84.6% accuracy and an area under the curve of only 0.897. For similar BAP1 mutation status with 54 samples, Feng et al. [51] reported an accuracy of 83% for Jackknife testing using Random Forest. Using image data for clear cell renal cell carcinoma, Acosta et al. proposed a deep-learning-based method for analyzing intertumoral heterogeneity and considered the three most frequently mutated genes, which were BAP1, PBRM1, and SETD2. Overall, the authors achieved an area under the receiver operating characteristic curve of around 0.89.

By considering the importance of clear cell renal cell carcinoma, Chen et al. [52] proposed a deep learning algorithm for the prediction of prognosis and immunotherapeutic response. The authors used data from 3 different cohorts, and samples were around 730 after performing pre-processing. After training the deep learning model for 100 epochs, the authors achieved a sensitivity of 0.71 and a specificity of 0.68.

The proposed method of the present study is trained on 10706 genes and 27685 instances, from which 1513 are other tumor drivers, 1272 are RCCC tumor drivers, and the rest are passenger gene mutation instances. The proposed system covers all the kidney genes and a huge number of tumor driver mutations, so it gives more accurate and reliable results after deployment.

## 5. Conclusions

The kidney is one of the vital organs in the human body, as it is responsible for blood cleaning, removing waste and poisonous substances from the blood, and balancing the electrolytes in the body. Kidney cancer is the most popular cancer in developing countries because of a lot of reasons, one of which is the huge amount of alcohol consumption. In this research work, a machine-learning-based efficient automated method is introduced for the prediction of kidney cancer before the development of kidney cancer. For this purpose, the proposed approach maintains the record of cancer driver mutations in the human body, and for this reason, the statistical position sensation calculation is performed. It then validates the approach with different types of testing techniques, which are Jackknife, independent dataset, and cross-validation. The Jackknife, cross-validation, and independent test accuracies of the system are 100%, 98%, and 83%, respectively.

## 6. Limitations and Future Work

The proposed RCCC_Pred system could be improved further in the future, and this work can be extended towards the betterment of the system to improve human health and to save more human lives, which are the targeted critical issues to make the system more perfect and updated. In the future, this work can also help develop AI systems for other human diseases, especially different kinds of cancers in other human body organs and tissues. Moreover, by increasing the number of data samples and employing deep neural networks, the method could be improved further in terms of performance.

## Figures and Tables

**Table 1:** Information of Positive and Negative Data for Classes.

Class	Positive Data	Negative Data
RCCC (kindey_tumour_driver)	RCCC (kindey_tumour_driver)	Other Tumor (other_tumour_driver) + Unknown (non-tumor driver)
Other Tumor (other_tumour_driver)	Other Tumor (other_tumour_driver)	RCCC (kindey_tumour_driver) + Unknown (non-tumour driver)
Unknown (non-tumour driver)	Unknown (non-tumour driver)	RCCC (kindey_tumour_driver) + Other Tumor (other_tumer_driver)

**Table 2:** Training accuracy results through self-consistency test.

Specificity	Sensitivity	Accuracy	MCC Stability
1.00	1.00	1.00	1.00

**Table 3:** Mean scores for 10-fold cross-validation results.

Specificity	Sensitivity	Accuracy	MCC Stability
0.99	0.97	0.98	0.96

**Table 4:** Mean scores for Jackknife validation results.

Specificity	Sensitivity	Accuracy	MCC Stability
0.98	0.99	0.99	0.99

**Table 5:** Comparison of the proposed method with other techniques.

Methods	Classifiers	Specificity	Sensitivity	Accuracy	MCC Stability
Kocak et al., 2019 [22]	ANN	87.8%	87.8%	88.2%	0.763
Kocak et al., 2019 [22]	Random Forest	94.6%	94.6%	95%	0.900
Proposed method	ANN + Hist Gradient boosting	98.3%	99.4%	98.87%	0.990

**Table 6:** Scores for Independent dataset testing of the proposed method.

Specificity	Sensitivity	Accuracy	MCC Stability
0.84	0.82	0.83	0.80

**Table 7:** Comparison of accuracy score for the proposed model with other ML algorithms.

Classifiers	Independent Test	Cross-Validation	Jackknife Test
Random Forest	0.82	0.97	0.86
Decision Tree	0.84	0.96	0.93
Naive Bayes	0.36	0.48	0.40
Gradient Boosting	0.80	0.96	1.00
Hist Gradient Boosting	0.83	0.98	1.00

## Data Availability

The dataset is provided as Supporting Information S1.

## Supplementary Materials

The following supporting information can be downloaded at: https://www.mdpi.com/article/10.3390/diagnostics12123036/s1.
